# A New Breath: Dynamics of Respiratory Infections After the Lifting of Non-Pharmaceutical Interventions Related to COVID-19

**DOI:** 10.3390/microorganisms13122710

**Published:** 2025-11-27

**Authors:** Rouba Keyrouz, Bassem Habr, Marianne Antar Soutou, Sirine Abou Ismail, Marianne Abifadel, Josette Najjar-Pellet, Bernard Gerbaka, Elie Haddad

**Affiliations:** 1 Infectious Disease Department, Faculty of Medicine, Saint Joseph University, Hôtel-Dieu de France, Beirut 1107 2180, Lebanon; 2 Pulmonary and Critical Care Department, Faculty of Medicine, Saint Joseph University, Hôtel-Dieu de France, Beirut 1107 2180, Lebanon; bassem.habr@usj.edu.lb; 3 Laboratoire Rodolphe Mérieux Liban, Faculty of Pharmacy, Saint Joseph University, Beirut 1004 2020, Lebanon; marianne.antar@usj.edu.lb (M.A.S.); sirine.abouismail1@usj.edu.lb (S.A.I.); marianne.abifadel@usj.edu.lb (M.A.); 4 Fondation Mérieux, Beirut 1100 2050, Lebanon; josette.najjar@fondation-merieux.org; 5 Pediatrics Department, Faculty of Medicine, Saint Joseph University, Hôtel-Dieu de France, Beirut 1107 2180, Lebanon; bernard.gerbaka@usj.edu.lb

**Keywords:** non-pharmaceutical interventions (NPIs), viral epidemiology, respiratory infection, virus, multiplex PCR, seasonal variation, coinfection, COVID-19, signs and symptoms, viral interference

## Abstract

The epidemiology of respiratory viruses shifted considerably following the COVID-19 pandemic and the subsequent rollback of non-pharmaceutical interventions (NPIs). The initial global containment strategies implemented during the SARS-CoV-2 outbreak profoundly altered viral transmission dynamics and circulation patterns. As the World Health Organization (WHO) declared COVID-19 no longer a public health emergency in May 2023, viral circulation began reverting to pre-pandemic trends. This retrospective observational study examined the evolving epidemiological patterns of respiratory infections during and after the lifting of NPI, assessing associated clinical manifestations and their relationship with patient-specific risk factors. Data were collected from 307 patients tested between October 2021 and December 2024 using a respiratory multiplex PCR at the Rodolphe Mérieux Laboratory in Lebanon. Results revealed a reemergence of pre-pandemic seasonal trends for most viruses. Rhinovirus remained the most prevalent pathogen, likely due to the absence of a vaccine. Respiratory syncytial virus (RSV) and Influenza A resumed their characteristic winter peaks, while human metapneumovirus (HMPV) showed no co-infections, suggesting viral interference. The persistence of Influenza A and SARS-CoV-2 appeared influenced by vaccine coverage, viral mutations, and environmental factors. Multiplex PCR testing proved to be a valuable yet costly tool for both diagnosis and epidemiological surveillance. Overall, this study highlights the importance of continued viral monitoring in the post-NPI period, reflecting both the effectiveness of NPIs in limiting viral spread and the importance of ensuring wider access to advanced diagnostic methods.

## 1. Introduction

COVID-19 is no longer classified as a “public health emergency of international concern” by the World Health Organization (WHO) as of May 2023; however, the disease is still considered a pandemic [1]. Based on current information, an official declaration marking its end is not anticipated in the near future [2]. The downturn in the SARS-CoV-2 pandemic largely followed the rollout of vaccines, which significantly reduced COVID-related adverse outcomes. There was a peak vaccine effectiveness of 51% against hospitalizations and 68% against acute illness [3]. And an overall protection in adults aged 65 years old and more against COVID-19–associated hospitalization was 45–46% in immunocompetent patients and 40% in immunocompromised patients [4].

Early in the pandemic, simple epidemiological models were sufficient to project case numbers. However, with changes in public behavior and reduced surveillance efforts, achieving accurate modeling has become more challenging. During the peak of the COVID emergency, strong surveillance enabled the rapid identification of new variants such as Omicron and allowed for timely responses [5]. Public health measures implemented during the pandemic—including masking, social distancing, and travel restrictions—also substantially reduced the circulation of other respiratory pathogens [6].

As these non-pharmaceutical interventions (NPIs) were gradually lifted, many respiratory viruses began to recirculate more widely. In addition to changes in population behavior, the phenomenon of viral interference may have influenced these dynamics: infection with one virus can induce a transient antiviral state in the host, temporarily reducing susceptibility to other viral infections [7].

While modeling has helped us to estimate the proportion of the population affected by SARS-CoV-2, studies have also identified individual characteristics that increase the risk of severe illness or death. These include the severity of symptoms, the evolution of vital signs [8], and established demographic risk factors [9] like age, sex, comorbidities, and the presence or absence of an underlying atopic status [10]. Such factors are crucial considerations when analyzing the symptoms and patterns of circulating respiratory viruses.

Assessing how the relaxation of NPIs has influenced the epidemiology of respiratory viruses is crucial for refining public health strategies and optimizing clinical management. To date, no study has specifically examined how the lifting of public health measures influenced viral circulation, clinical presentation, and patient characteristics within the Lebanese population. To address this gap, we conducted a retrospective observational study of 307 patients tested between 2021 and 2024 at the Rodolphe Mérieux Laboratory in Beirut, Lebanon, using respiratory multiplex PCR. The study aimed to compare the epidemiological and clinical characteristics of respiratory infections between the NPI period (October 2021–May 2023) and the post-NPI period (May 2023–December 2024), examining infection patterns, symptom profiles, and their associations with demographic and individual risk factors.

## 2. Methods

### 2.1. Study Design and Population

It is a retrospective observational study spanning four years (2021–2024), based on data from computerized patient records and the results of the BIOFIRE Respiratory Panel 2.1 *plus* (RP2.1*plus*) performed at the Rodolphe Mérieux Laboratory (LRM) in Beirut, Lebanon. The study included a total of 307 patients who underwent multiplex PCR testing at the LRM between 2021 and 2024. The NPI period covered October 2021 to May 2023, followed by the post-NPI period from May 2023 to study completion. Overall, 108 valid respiratory panel tests were performed prior to 1 May 2023, and 199 during the post-NPI period.

### 2.2. Ethical Considerations

The study was approved by the Ethics Committee of the Saint Joseph University of Beirut (#CEHDF2273).

### 2.3. Respiratory Pathogen Detection with BioFire^®^ FilmArray^®^ RP2.1plus Panel

Respiratory specimens were analyzed at the Rodolphe Mérieux Laboratory using the BioFire^®^ FilmArray^®^ Respiratory Panel 2.1 *plus* (BioFire Diagnostics, bioMérieux, Marcy l’Etoile, France), a multiplex nested PCR assay designed for the qualitative detection of viral and bacterial pathogens directly from clinical samples.

The panel simultaneously targets 19 viral (Adenovirus, Coronavirus 229E, Coronavirus HKU1, Coronavirus NL63, Coronavirus OC43, Severe cute respiratory syndrome coronavirus 2 or SARS-CoV-2, Human metapneumovirus, Human rhinovirus/enterovirus, Influenza A(including subtypes H1, H3, and H1-2009, Influenza B, Parainfluenza virus 1, Parainfluenza virus 2, Parainfluenza virus 3, Parainfluenza virus 4, Respiratory syncytial virus) and 4 bacterial respiratory pathogens (Bordetella pertussis, Bordetella parapertussis, Chlamydophila pneumoniae, and Mycoplasma pneumoniae).

Sample processing and testing were performed according to the manufacturer’s instructions. Briefly, 300 µL of nasopharyngeal swab specimens collected in viral transport medium (VTM) were mixed with the provided sample buffer and loaded into the FilmArray RP2.1*plus* pouch. The pouch was then inserted into the FilmArray 2.0 instrument (bioMérieux), which performs automated nucleic acid extraction, reverse transcription, nested multiplex PCR amplification, and detection using melting curve analysis. The total runtime per assay was approximately 45 min, with results automatically generated and reported by the system software. Positive and negative internal controls included in each pouch ensured assay validity. Results were interpreted qualitatively (detected/not detected) for each target pathogen.

### 2.4. Data Collection

Clinical and demographic data were extracted from electronic patient records. Variables included age, sex, cardiovascular risk factors, history of asthma or atopic predisposition, other chronic comorbidities, immunosuppression or pregnancy status, and presenting clinical symptoms.

### 2.5. Statistical Analysis

Statistical analysis was performed using SPSS^®^ version 30.0.0 and Microsoft Excel^®^ 365 version 16.93. Descriptive statistics were used to assess the prevalence of respiratory infections by age and sex, and to identify the most common clinical symptoms. The frequency of multiplex PCR tests was compared by season and by month. The distribution of respiratory pathogens was analyzed to determine viral prevalence, including co-infections involving two or more pathogens. Chi-square tests were used to evaluate viral distribution by season, month, the presence of a concomitant co-infection, and clinical symptoms in relation to PCR results.

## 3. Results

### 3.1. Baseline Characteristics of the Study Population

**Figure 1:**
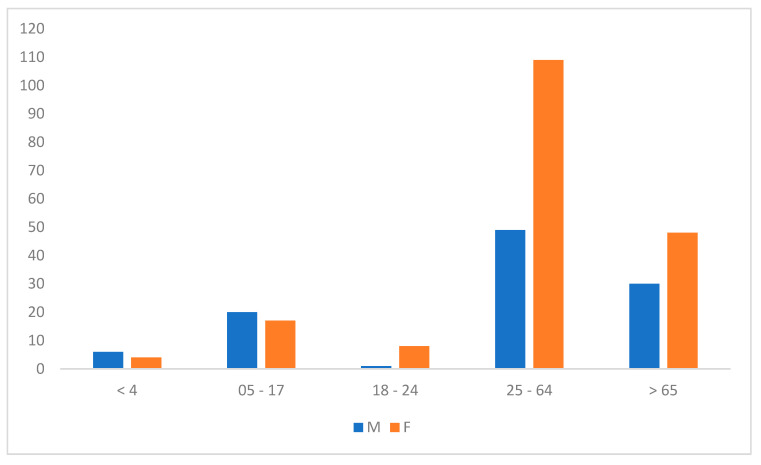
Demographic distribution of multiplex PCR tests across sex and age groups (years) (M: Male; F: Female).

A total of 307 patients were included in this study. Age data was missing for 15 patients. Among the remaining participants, most (51.5%) were aged between 25 and 64 years (Figure 1), with a female to male sex ratio of 0.57.

### 3.2. Annual, Seasonal and Monthly Variations in Samples

**Figure 2:**
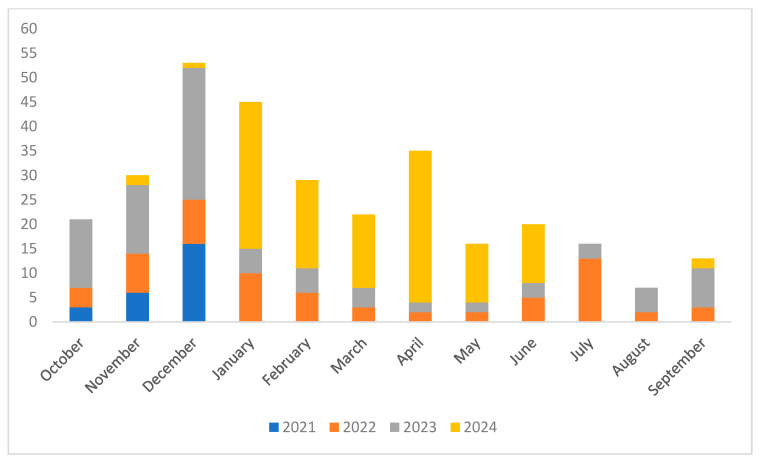
Monthly variation in multiplex PCR test activity from 2021 to 2024.

A clear and progressive increase in the number of tests performed was observed from 2021 to 2024, rising from 10% of the total tests in 2021 to 40% in 2024. Regarding seasonal variation, the highest number of tests were performed during winter (102 cases), followed by spring (71 cases), summer (43 cases), and fall (40 cases). However, chi-square analysis revealed no statistically significant differences in PCR testing across the seasons, suggesting that clinical triage by physicians remained consistently applied throughout the year. On a monthly scale, a prominent peak in testing occurred in December (18.2%), with secondary elevations in January (14.6%) and April (13.6%), as shown in Figure 2. Testing frequency progressively increased from September through December, followed by a decline in January. The peak observed in April may reflect increased viral circulation during atypical (off-season) periods.

### 3.3. Monthly and Seasonal Distribution of Multiplex PCR Testing and Corresponding Positivity Rates

**Figure 3:**
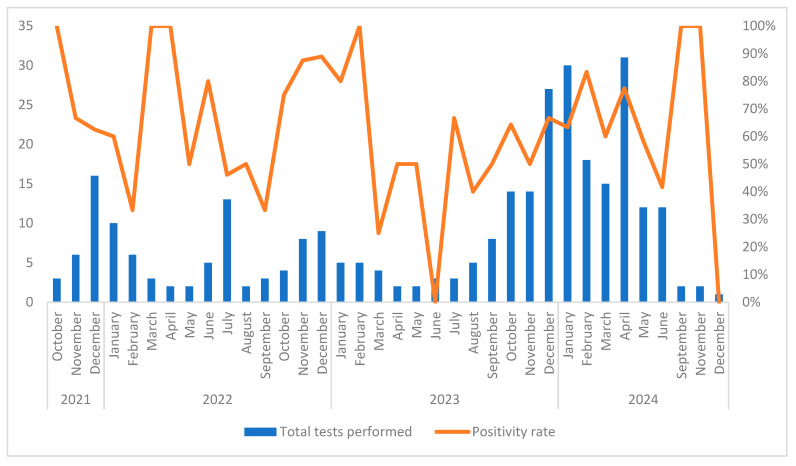
Monthly distribution of multiplex PCR tests performed and corresponding positivity rates between 2021 and 2024.

The data from late 2021 to the end of 2024 show clear variations in both testing volume and positivity rates (Figure 3). Months with very few tests often showed 100% positivity, particularly in October 2021, early 2022 (March, April), and February 2023. In contrast, the number of tests increased significantly during 2023 and 2024, reaching the highest level in April 2024 with 31 tests performed.

**Figure 4:**
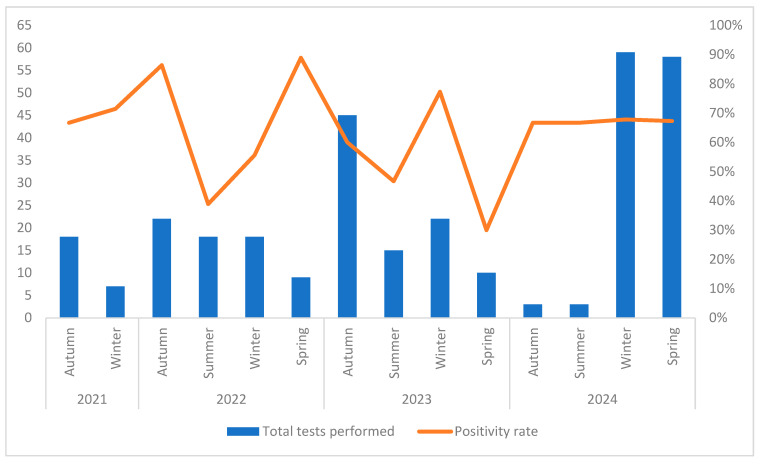
Seasonal distribution of multiplex PCR tests performed and corresponding positivity rates between 2021 and 2024.

When results were grouped by respiratory season (October to September), clear temporal patterns emerged (Figure 4). The positivity rate declined during the warmer periods of 2022 and 2023, while pronounced peaks were observed during the colder months, particularly in autumn, winter and spring 2022, winter 2023, and again in autumn 2024. The 2023–2024 season also showed the highest testing activity, with a total of 175 multiplex PCR tests conducted.

### 3.4. Distribution of Respiratory Pathogens

**Figure 5:**
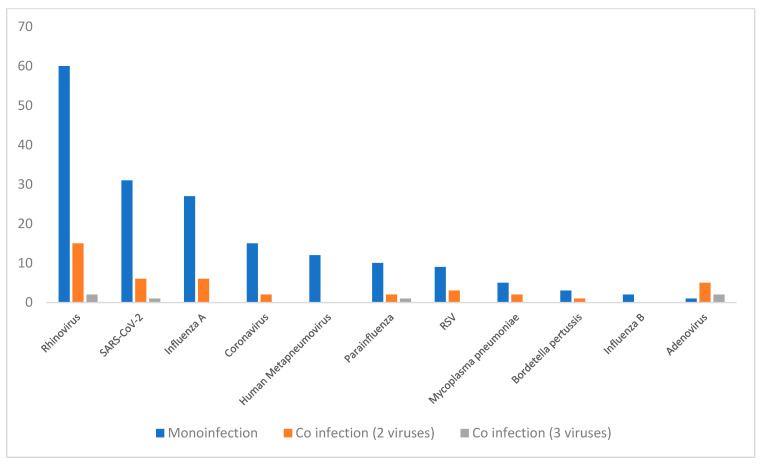
Frequency of respiratory pathogens.

Among the 307 patients, 198 (64.5%) tested positive for at least one pathogen by PCR, while 109 (35.5%) tested negative. Of the positive cases, 175 patients (57%) had a mono-infection. Co-infections were found in 23 patients (7.5%) of whom 21 (6.8%) had two pathogens, and 2 (0.7%) had three pathogens. The distribution of viral infections, including both mono-infections and co-infections, is shown in Figure 5.

### 3.5. Co-Infection Analysis

Among patients with co-infections, Rhinovirus was the most frequently detected virus, aligning with its overall predominance in the study population. Notably, 20% of all Rhinovirus infections occurred alongside another pathogen. As for Adenovirus, although it was detected in a small number of patients, it had a high co-infection rate, with 87.5% of cases involving concomitant coinfection. In contrast, no co-infections were observed in patients infected with Human Metapneumovirus (HMPV) (Figure 5).

### 3.6. Pre/Post Rate Ratios

**Figure 6:**
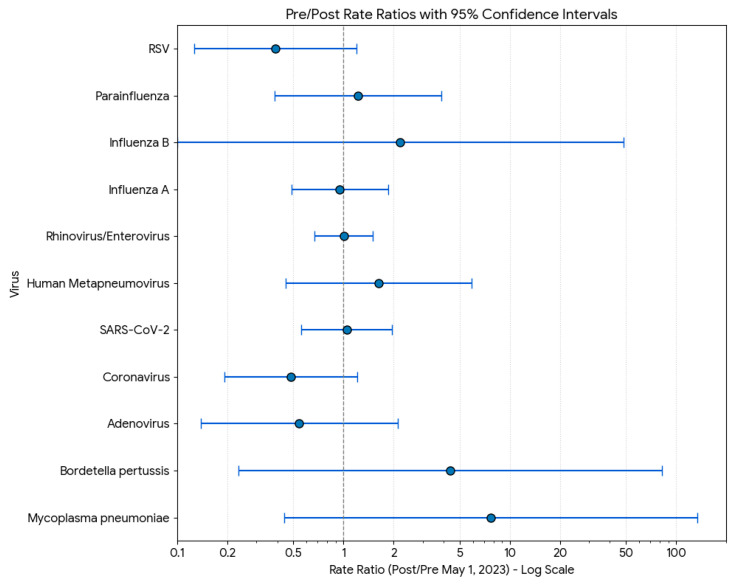
Pre and post rate ratios of viral circulation.

The analysis of the pre/post rate ratios for each virus, using a cutoff date of 1 May 2023, is summarized in Figure 6. The rate ratio (RR) is calculated as the proportion of positive tests in the post-period (1 May 2023, onwards) divided by the proportion in the pre-period (before 1 May 2023). The wide confidence intervals for *Mycoplasma pneumoniae* and *Bordetella pertussis* are due to the very low (zero) number of positive cases in the pre-period, leading to higher uncertainty in the rate ratio estimation. The analysis, using 1 May 2023 as the cutoff, revealed varied changes in the proportion of positive respiratory virus tests between the pre- and post-periods. Only two viruses showed a statistically significant change at the 95% confidence level: the rate for RSV (RR = 2.59) increased significantly in the post-period, while the rate for Influenza A (RR = 0.19) decreased significantly. The remaining nine viruses showed no statistically significant change, as their 95% confidence intervals crossed the null value of Rate Ratio = 1. While Mycoplasma pneumoniae (RR = 7.67) and Bordetella pertussis (RR = 4.38) had the largest point estimates for an increase, the wide margins of their confidence intervals—a result of low counts in the pre-period—indicate that these changes were not statistically significant and are associated with high uncertainty. Similarly, viruses like Rhinovirus/Enterovirus (RR = 0.28), Coronavirus (RR = 0.48), and Adenovirus (RR = 0.54) all trended toward lower rates post-cutoff, but their changes also fell within the range of statistical non-significance.

### 3.7. Correlation Between Viral Infections and Symptoms

**Figure 7:**
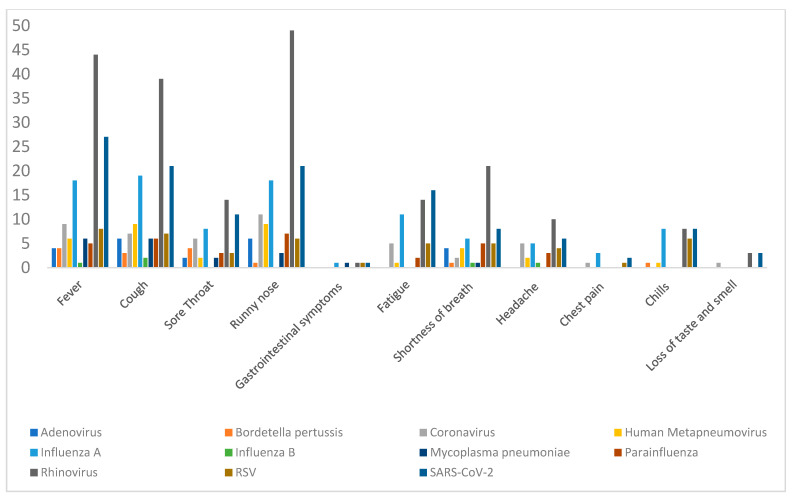
Distribution of clinical symptoms across major respiratory pathogens.

Among 307 patients, 198 tested positive for at least one virus, yielding 223 positive results when accounting for co-infections. Nearly half of these patients (45.9%) had at least one underlying comorbidity. Rhinovirus was the most prevalent (25.1%, 77 patients), followed by SARS-CoV-2 (12.4%, 38 patients) and influenza A (10.7%, 33 patients); all other viruses were detected in fewer than 30 patients. Fever was the most commonly reported symptom (157 patients, 51.1%), followed by rhinorrhea (152 patients, 49.5%) and cough (145 patients, 47.2%) (Figure 7). Overall, clinical presentations were similar across viral infections, with few statistically significant associations. Fatigue was significantly associated with Influenza A and SARS-CoV-2 infections, as supported by both Pearson’s chi-square test (*p* = 0.022 for Influenza A; *p* = 0.01 for SARS-CoV-2) and the likelihood ratio test (*p* = 0.03 for both viruses). A potential association between dyspnea and other human coronaviruses was suggested by the likelihood ratio test (*p* = 0.03); however, the asymptomatic two-sided significance was 0.1, indicating limited statistical strength, likely due to small sample size. Similarly, trends in headaches did not reach statistical significance. Other less frequent symptoms included chest pain (10 patients) and anosmia or ageusia (8 patients). Among those reporting anosmia or ageusia, 3 were infected with SARS-CoV-2, 1 with another coronavirus, and 3 with Rhinovirus. Statistical analysis was not feasible for these symptoms due to the small numbers. Collectively, Figure 7 illustrates the distribution of clinical symptoms across the major respiratory pathogens, highlighting which virus was most frequently associated with each symptom.

### 3.8. Local Viral Epidemiology

**Figure 8:**
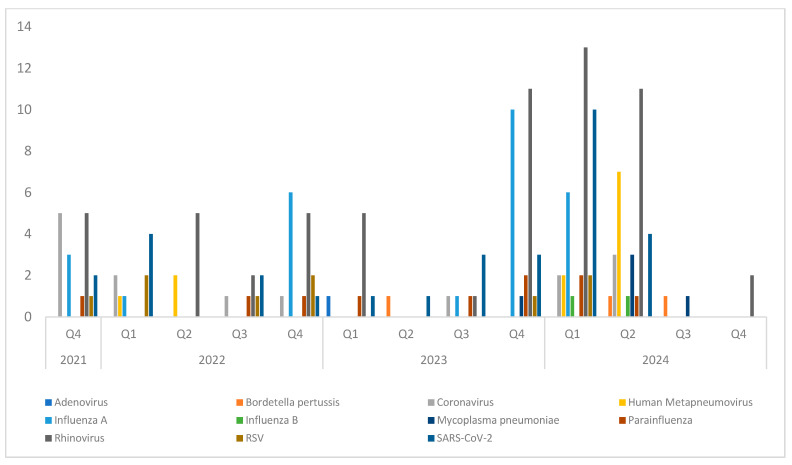
Quarterly distribution of respiratory pathogens from 2021 to 2024.

**Figure 9:**
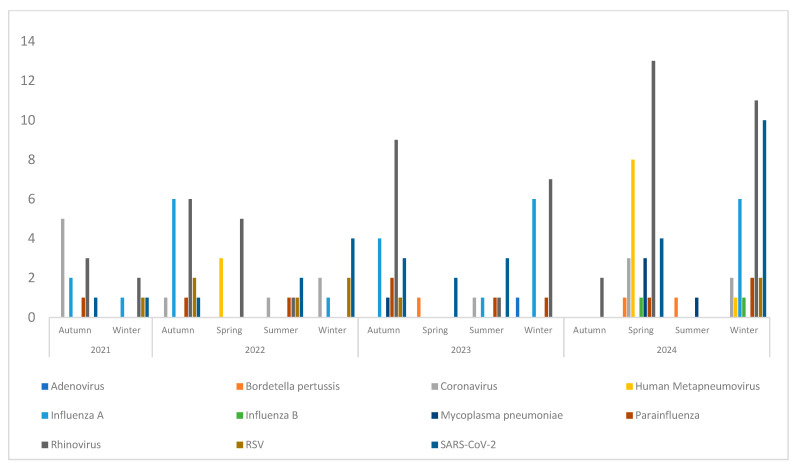
Seasonal distribution of respiratory pathogens from 2021 to 2024.

Changes in local respiratory virus circulation from 2021 to 2024 are shown in Figure 8 and Figure 9, reflecting trends during and after the lifting of COVID-19 restrictions. Rhinovirus showed little variation between years, consistent with its endemic nature. SARS-CoV-2 remained detectable throughout the study period, with minor fluctuations, though it was no longer the predominant virus. Influenza viruses continued to exhibit seasonal peaks, with variability in the magnitude of each year’s peak. To examine seasonal patterns in more detail, viral trends were analyzed by quarter. Quarterly analysis (Figure 8) shows two clear peaks of Influenza A during the fourth quarters of 2022 and 2023, consistent with its established winter seasonality. Seasonal human coronaviruses (excluding SARS-CoV-2) also showed increased circulation, suggesting a return to pre-pandemic patterns. HMPV activity was observed in the second quarters of 2022 and 2024, in line with its typical late winter to early spring seasonality. Seasonal trends by broader periods are also shown in Figure 9, providing an overview of virus circulation across each season.

## 4. Discussion

This research builds upon the study conducted by Manuela Avolio et al. [11], which analyzed viral circulation patterns from 2018 to 2021. Their work established the pre-pandemic and pandemic landscape of respiratory viruses during the implementation of COVID-19 public health interventions. Specifically, Avolio et al. reported the unprecedented suppression of most seasonal respiratory viruses. Our current findings, therefore, complement and extend their dataset by covering the subsequent post-pandemic period (2021–2024).

This extension provides new insights into the recovery of pre-pandemic viral epidemiology. With the easing of social distancing and other restrictions, seasonal viruses such as influenza and RSV reemerged, often displaying altered timing, magnitude, and circulation patterns. Thus, while the study by Avolio et al. documented the unprecedented suppression of respiratory viruses, our research illustrates their rebound and the re-establishment of balance within the respiratory viral ecosystem.

The COVID-19 pandemic and the resulting public health interventions radically reshaped the epidemiology of common respiratory viruses. From 2018 through 2021, there was a progressive decline in overall respiratory viral circulation, with nearly all seasonal viruses reaching near-suppression levels following the implementation of strict control measures such as mask-wearing, social distancing, and lockdowns. Rhinovirus was the only virus to show an unmistakable pattern of resilience and persistence during this interval. While most other respiratory viruses either disappeared or were strongly suppressed, Rhinovirus continued to circulate, maintaining its characteristic winter peaks. This consistent activity made rhinovirus the most frequently detected virus, surpassing even SARS-CoV-2 in certain settings. Its sustained circulation can be attributed to several factors: the absence of a vaccine or effective antiviral therapy, its typically mild clinical presentation leading to fewer diagnostic restrictions, and its high infectivity and environmental stability. Furthermore, its ongoing dissemination was likely facilitated by the gradual relaxation of public health measures over time, which increased interpersonal contact and marked a return toward pre-pandemic social interaction patterns, thereby enabling Rhinovirus transmission [11].

Our study corroborates this pattern and further demonstrates the re-establishment of typical seasonal peaks for Respiratory Syncytial Virus (RSV) in November–December and Influenza A in December. These findings align with previous literature linking viral seasonality to meteorological conditions [12]. The evidence confirms the observation that respiratory virus circulation has largely reverted to pre-pandemic levels.

Human Metapneumovirus (HMPV) and Respiratory Syncytial Virus (RSV) are two viruses within the Pneumoviridae family which are very closely related. They co-circulate over the winter and spring months, with circulation of HMPV typically following the peak of the RSV season. While our data confirmed their seasonal presence, none of the eight patients infected with HMPV were co-infected with RSV. The literature remains inconclusive on this specific virus-virus interaction, with some studies reporting very high co-infection rates—up to 70% in certain severe cases—while others, much like our own findings, suggest limited co-detection [13,14].

Seasonality of Influenza A remains a constant feature in most regions, with its peak occurrence generally in winter, most commonly in December and January. This very prominent winter peak may be due to a number of interconnected elements, extending from the issue of low vaccine coverage to the yearly complication of antigenic mismatch between the vaccine strains selected and the circulating viruses [15]. In locations such as Lebanon, suboptimal vaccine coverage also contributes to such vulnerability, with a significant proportion of the population left susceptible [16]. Similarly, SARS-CoV-2, having been a risk of pandemic, has now come to the endemic phase when it continues to spread among humans in spite of mass vaccination programs [13,17]. The continued prevalence, or even re-emergence, of infection by SARS-CoV-2 can, to a large degree, be ascribed to the continued identification of novel variants [17]. These emerging strains often possess mutations with partial immune escape, which allows them to circumvent the immunity conferred by past infection or existing vaccines, despite high vaccine coverage [17]. Beyond immunological and viral evolution, environmental determinants are at the core of the winter transmission cycles for both viruses. In particular, the winter season brings about increased indoor crowding as people remain indoors for longer periods, creating an ideal setting for close contact aerosol and droplet transmission. Moreover, high humidity—that may be a factor in certain indoor environments or in particular weather conditions—or low humidity, respectively, may affect viral particle stability and infectivity, further fostering viral transmission during the winter season [18].

The relatively high rate of co-infection with Adenovirus, even in a small population size, is an interesting observation that implies viral synergy. This may biologically account for the unexpected prevalence of co-detection, as the composite infection might lead to a more severe clinical presentation to warrant outpatient doctor visit and PCR testing or hospitalization. In fact, research has revealed that co-infection of Human Adenovirus with other viruses or bacterial/fungal disease is associated with increased risk of severe clinical presentation in children compared to mono Adenovirus infection, lending credence to the synergy hypothesis [19].

Conversely, the absence of co-infections in patients with Human Metapneumovirus (HMPV) could hypothetically point toward a viral interference (antagonistic) mechanism. Viral interference is a negative interaction where the presence of one virus inhibits the infection or replication of a second virus. The most probable mechanism is the rapid and robust induction of a non-specific innate immune response, by the first virus, which creates a temporary, broadly antiviral state in the host cells, blocking the second virus [7].

The COVID-19 pandemic introduced additional complexity, as non-pharmaceutical interventions (NPIs) such as masking and social distancing disrupted traditional seasonality, leading to atypical circulation patterns and delayed resurgence of winter viruses. Subsequent relaxation of NPIs contributed to immunity gaps and out-of-season activity for some pathogens, notably RSV and influenza. These findings underscore the interplay between environmental conditions, human behavior, and public health measures in shaping respiratory virus seasonality. Understanding these dynamics is critical for optimizing surveillance and timing interventions such as vaccination campaigns and prophylaxis [20,21].

Given the increased healthcare burden of respiratory infections, future research will have to clarify the clinical symptoms most correlated with healthcare utilization or hospitalization. Comparative within-virus and between-virus virulence analyses, as well as larger cohorts, will be required to examine whether viral interactions such as synergy or interference are contributing to co-circulation patterns [7].

Most importantly, the application of multiplex PCR enhanced this study even further. This diagnostic instrument enabled the detection of several different respiratory pathogens from one single sample and provided a quick, standardized, sensitive, and specific approach. Increased application of multiplex PCR would enhance epidemiologic surveillance on a large scale [7].

Importantly, the application of multiplex PCR significantly strengthened this study. This diagnostic modality enabled simultaneous detection of multiple respiratory pathogens from a single sample and provided a rapid, standardized, sensitive, and specific approach. Wider implementation of multiplex PCR would enhance large-scale epidemiological surveillance.

However, the study had several limitations, the first being the study sample size and variability in referral patterns. This study was limited to a total of 307 patient samples. The referral process was not uniform; while some patients underwent testing without prior clinical investigation, others were referred for multiplex PCR following negative rapid test results for influenza and/or SARS-CoV-2. Most patients were assessed in ambulatory care settings; however, a minority were hospitalized, with samples submitted through corresponding hospital departments. All samples were retained to strengthen the epidemiological analysis. It is important to note that incomplete medical records for some patients has constrained the ability to conduct detailed analyses exploring associations between clinical symptoms and potential risk factors.

The second limiting factor was the study period, as the data collection period ended in late December 2024. Following this cutoff, the number of patient referrals to the laboratory declined considerably, likely due to a shift in testing practices, with many individuals seeking influenza and COVID-19 diagnostics at alternative centers. This timing meant our study missed a notable surge in influenza activity that occurred in January and February 2025.

This study was conducted within a single-center laboratory serving a mixed population of outpatients and hospitalized patients, with uneven referral patterns. Such a setting introduces potential selection bias, as the tested population may not accurately represent the broader community or other healthcare environments.

Extending the duration of data collection and increasing the overall sample size would enhance the accuracy of outbreak characterization and improve the robustness of seasonal trend analyses.

Finally, although PCR multiplex testing is extremely sensitive and specific, limited national access to such tests remains a restriction to expansive epidemiological surveillance. Greater access to these testing technologies will facilitate improved detection and surveillance of respiratory viruses in circulation between varied populations.

## 5. Conclusions

Following the gradual relaxation of containment measures implemented during the COVID-19 pandemic, we observed a resurgence in the circulation of respiratory viruses—most notably Rhinovirus and Influenza A—alongside persistent SARS-CoV-2 activity. This resurgence aligns with pre-pandemic seasonal epidemiological patterns.

Respiratory multiplex PCR proved to be a valuable diagnostic tool, particularly in patients with negative rapid antigen tests. Its sensitivity and its rapidity offer significant advantages for clinical and epidemiological use.

Our data revealed notable monthly variation in testing frequency, with a distinct peak in December (18.2%) and additional elevations in January (14.6%) and April (13.6%). The April peak may indicate off-season viral activity.

Among positive cases, 57% represented mono-infections, of which: Rhinovirus was the most frequently detected virus in coinfections, appearing in 20% of such cases—consistent with its overall prevalence. Although only a small number of patients tested positive for adenovirus, 87.5% of these cases were coinfections, possibly reflecting viral synergy. In contrast, none of the HMPV-positive patients were coinfected, suggesting potential viral interference.

## Data Availability

The original contributions presented in this study are included in the article. Further inquiries can be directed to the corresponding author.
